# Consumer Preferences of Topical Vitamin C Products: A Comparative Study

**DOI:** 10.7759/cureus.45414

**Published:** 2023-09-17

**Authors:** Michelle Lazar, Susruthi Rajanala, Henriette De La Garza, Neelam Vashi

**Affiliations:** 1 Dermatology, Boston University Chobanian & Avedisian School of Medicine, Boston, USA

**Keywords:** dermatology trends, cosmetics, consumer preferences, ascorbic acid, vitamin c, hyperpigmentation

## Abstract

Hyperpigmentation disorders greatly impact the quality of life of many patients. Vitamin C and ascorbic acid derivatives are commonly used as topical agents to reduce the effect of facial dyschromia. The market for vitamin C topicals has greatly increased, but with little oversight, resulting in a wide variety of products with regard to purity and efficacy. In this study, we conducted an analysis of the most highly rated vitamin C-containing compounds on Amazon and Sephora. It was found that consumers for both retailers prioritized efficacy and cosmetic elegance in their rating of products. Additionally, consumers displayed no preference among vitamin C derivatives in the products available to them. This study highlights the diverse vitamin C formulations currently available as well as consumer preferences, emphasizing the importance of fully reviewing all products patients are utilizing to better counsel on treatment regimens.

## Introduction

Facial hyperpigmentation disorders have a significant impact on quality of life, a finding that is echoed across cultures [1,2]. Hyperpigmentation disorders are a common cause of physician-patient interactions and can be particularly distressing to patients due to the visible nature of the disease [3]. In particular, hyperpigmentation disorders have been shown to have a disproportionately negative impact on skin of color patients, in whom pigmentation can be stubborn and difficult to treat [4]. These treatments are often associated with a high cost and additionally, many patients prefer treating themselves at home with over-the-counter (OTC) options. This, in turn, drives demand for OTC skincare products or cosmeceuticals aimed to enhance skin brightness and reduce hyperpigmentation [5,6]. This is a rapidly growing market that is both poorly regulated and non-standardized and cosmeceuticals are able to make claims without evidence or data to support these claims as they are not regulated by the FDA [7].

Recently, there has been rising consumer interest in vitamin C serums, shown by both growing sales and an increase in Google searches for these products [8,9]. Several vitamin C derivatives exist on the market, and products must be formulated carefully to allow efficient penetration into the skin and prevent the breakdown of vitamin C [5]. While it is well known that ascorbic acid has one of the highest bioavailability of all forms of vitamin C, it is not the most effective agent due to its hydrophilicity, which makes penetration of the stratum corneum less efficient than other derivatives [5]. Additionally, ascorbic acid in high concentrations can be irritating to those with sensitive skin, and it has been shown that concentrations over 20% do not increase the effect of the ascorbic acid delivered [10]. As a result, many products currently on the market use other derivatives of vitamin C, including L-ascorbic acid, magnesium ascorbyl phosphate (MAP), 3-O-ethyl ascorbic acid (EAC), and tetrahexyldecyl ascorbate (THDA) [5]. The many derivatives vary in terms of their stability and their ability to inhibit melanogenesis via tyrosine inhibition. For instance, while L-ascorbic acid has strong data suggesting it inhibits melanogenesis via tyrosine inhibition, there are no data yet suggesting that the same can be said of ascorbyl-6-palmitate [5]. Therefore, while all products may be marketed for the same use and contain many of the same active ingredients, their effects can be greatly altered by the additional compounds in the formula.

The most efficacious mechanism of vitamin C delivery has not been ascertained as of yet. A systematic review on microneedling efficacy for melasma treatment found that the combination of vitamin C and microneedling led to a 3.7% decrease in the Melasma Area Severity Index (MASI), and while not the most dramatic of all studies found in the systematic review, still a considerable improvement from the patient’s baseline [11]. By generating small puncture wounds in the skin, microneedling allows vitamin C to bypass the stratum corneum and therefore helps increase the efficacy of the compound. However, there have been case reports that identify granuloma formation as a rare adverse event as a result of microneedling with vitamin C [12-14]. In these cases, systemic workup for sarcoidosis was negative, indicating that the reason for the granuloma formation was likely tied to the utilization of vitamin C serums along with the microneedling treatment [12-14]. This highlights the need for stringent assessment of compound formulation as well as any additives that may be found in serums used along with microneedling treatment, to offset the rate of this and other adverse effects. Other authors have endorsed the use of laser technology for transdermal drug delivery. It has been posited that lasers may increase the efficacy of vitamin C delivery by disrupting the skin to allow for better penetration of compounds [15].

Generally, vitamin C is one of the most commonly used OTCs for hyperpigmentation [1]. It can be found in a variety of forms and at a lower price point than alternative treatment options. This makes it easily accessible for many patients; however, given the multitude of options, consumers often have difficulty distinguishing between products and identifying which will have the best efficacy for their specific concerns. In this study, we aim to assess consumer preferences for topical vitamin C formulations by analyzing customer reviews of top-selling products to better understand what characteristics patients value most, and better characterize OTC topical vitamin C products.

## Materials and methods

In October of 2022, authors searched for the keyword “vitamin C serum” on Sephora.com and the “Beauty and Personal Care” category on Amazon.com. These sources were chosen to compare product availability and consumer preferences between an exclusive beauty retailer versus a large online retailer. Inclusion criteria included products with at least 100 reviews, products containing a vitamin C derivative, and products that were marketed for facial use. The top 20% of eligible products were selected by filtering for products with four-star or greater reviews, and then further narrowed to products with 100 reviews or more, with 41 Sephora products and 144 Amazon products included in the analysis. Data for all eligible products were collected and included formulation, vitamin C derivative, price, price per ounce, rating, and number of reviews. Duplicate products, products not marketed primarily as a vitamin C treatment, and products that were solely marketed for body and hair care were excluded. We then sampled the top five positive and top five most critical reviews for each product. Two authors (S.R. and H.D.L.G) then analyzed reviews and coded positive and negative descriptors into the following categories: cosmetic elegance, efficacy, and tolerability. The cosmetic elegance category comprised mentions of texture (e.g., greasy or slippery), ability to blend in the product, ability to layer with makeup, scent, and overall appearance of the product when applied on the skin. Efficacy was noted by mentions of how well the product worked to improve or fade dark spots or brighten the skin. Finally, comments on tolerability noted any irritation or adverse reactions experienced by consumers.

## Results

In total, 41 products and 410 reviews from Sephora and 144 products and 1440 reviews from Amazon were included in the data analysis (Table 1). Serums were the most common product formulations (n = 179). The average cost per ounce ($63.75 for Sephora vs. $25.11 for Amazon) and average number of reviews for products (456.5 for Sephora vs. 3034 for Amazon) between the two retailers were significantly different (two-tailed unpaired parametric T-test with Welch’s correction, p < 0.05). However, the average rating for products from Sephora vs. Amazon was not significantly different (two-tailed unpaired parametric T-test with Welch’s correction, p = 0.57).

**Table 1:** Study metrics Variables such as cost by ounce, rating, number of reviews, and others were analyzed for each product that met inclusion criteria on Sephora and Amazon. These values were then collated to create representative averages for each company. Statistical analysis was conducted by a two-tailed unpaired parametric T-test with Welch’s correction between values for Sephora and Amazon to identify key differences in the products offered by the retailers. Statistical analysis was conducted by a two-tailed unpaired parametric T-test with Welch’s correction between the active ingredient and average rating to identify if consumers had a preference for certain vitamin C derivatives.

Metrics	Sephora	Amazon	P-value, Sephora vs. Amazon
Average cost by ounce	$63.75	$25.11	<0.001
Average rating	4.39	4.37	0.5702
Average number of reviews	456.54	3033.54	0.0002
Cosmetic elegance, positive	84	251	-
Cosmetic elegance, negative	94	211	-
Effectiveness, positive	174	486	-
Effectiveness, negative	92	239	-
Tolerance, positive	42	86	-
Tolerance, negative	80	224	-

As the unpaired t-test was not significantly different between the two retailers, statistical analysis of active ingredients was not differentiated by retailer but rather by vitamin C derivative (Table 2). The most common active ingredient among all products was ascorbic acid (n = 66), followed by sodium ascorbyl phosphate (n = 58).

**Table 2:** Active ingredients The most commonly used forms of vitamin C on Sephora and Amazon, both by raw count and percentage of products. The average rating of products excludes products that had multiple forms of vitamin C in their ingredient list.

Active ingredients	Number of appearances, N (%)	Average rating (excluding overlapping products)
Ascorbic acid	66 (35.68%)	4.4
Sodium ascorbic phosphate	58 (31.35%)	4.35
3-O-ethyl ascorbic acid	25 (13.51%)	4.41
Magnesium ascorbyl phosphate	21 (11.35%)	4.35

After excluding products that included multiple vitamin C derivatives, our data show that there was no significant difference between active ingredients and average consumer rating (Table 3). For both Sephora and Amazon products, efficacy was the most cited positive feature in comments. Among negative comments, cosmetic elegance and efficacy were most frequently cited for Sephora and Amazon products, respectively.

**Table 3:** Active ingredient and consumer rating The average consumer rating between forms of vitamin C was compared. Statistical analysis was conducted by a two-tailed unpaired parametric T-test with Welch’s correction, none of the analyses were statistically significant.

Active ingredients	P-value, ___ vs. ascorbic acid, average rating	P-value, ___ vs. sodium ascorbyl phosphate, average rating	P-value, ___ vs. 3-O ethyl ascorbyl phosphate, average rating
Ascorbic acid	-	-	-
Sodium ascorbyl phosphate	0.1702	-	-
3-O-ethyl ascorbic acid	0.7533	0.2931	-
Magnesium ascorbyl phosphate	0.4624	0.9716	0.5049

The two highest-rated products received a 4.8 out of 5 stars rating. There was a nine-way tie for the third most highly ranked product among both Amazon and Sephora, with the remainder receiving a 4.7. Two of the products came from Amazon while nine came from Sephora. The two most highly rated products contained EAC or L-ascorbic acid as their active form of vitamin C. Among the top 11 products, six products contained ascorbic acid, two contained EAC, two contained ascorbyl tetraisopalmitate, one contained THDA, one contained MAP, one contained sodium ascorbyl phosphate, and one contained ascorbyl glucoside. The top five negative and positive comments were analyzed for each of the top 11 compounds. Of these products, the average score was 2.63/5 for the proportion of the product’s top five comments that mentioned cosmetic elegance and 3.45/5 for the proportion of the top 11 products' top five negative comments that mentioned cosmetic elegance. This indicated that for the top 11 products, cosmetic elegance was a strong factor in determining both negative and positive reviews. Additionally, of these top 11 products, the average score was 4.09/5 for the portion of the top 11 products' top five comments that mentioned effectiveness while only 2.55/5 of the top five negative comments mentioned effectiveness. This indicates that the effectiveness of a compound seemed to have a stronger impact in the positive reviews than the negative. Finally, the average score was 1.09/5 for the portion of the top 11 products' top five comments that mentioned tolerance and 1.64/5 for the portion of the top 11 products' top five negative comments that mentioned tolerance. This indicates that tolerance seems to have the least impact on reviewer scores in the top 11 products among the categories sampled.

## Discussion

Facial hyperpigmentation disorders, including melasma, post-inflammatory hyperpigmentation, solar lentigines, and maturational hyperpigmentation, are highly prevalent and a frequent presenting complaint to dermatologists, especially among the skin of color population. While exact pathophysiology varies among these etiologies, excess production of melanin plays a common role and offers a target for treatments [16]. In addition, it is known that facial hyperpigmentation can have a significant negative impact on patients’ psychological well-being and quality of life (QoL). One study assessing the impact of corrective makeup on improving QoL found that, on average, patients with pigmentary disorders, including melasma, were significantly more depressed compared to patients with acne and vascular disorders [17]. Another recent Indian study found that Skindex scores (a validated assessment tool to evaluate the impact on QoL) were overall high among patients with facial melanoses [18]. As a result, it is important to identify the best treatment methods for these patients to increase overall health outcomes and ensure patient satisfaction.

Vitamin C (ascorbic acid) compounds have quickly grown in popularity in OTC skincare products [8]. These products are sold as cosmeceuticals, as they have a cosmetic benefit, low risk profile, and are not regulated by the Food and Drug Administration [7]. This leads to large variability in both the purity and stability of current products on the market. Vitamin C interrupts copper ions, inhibiting the tyrosinase pathway, and thereby decreasing melanin synthesis. In addition, it also plays a role in collagen synthesis and serves as an antioxidant, making it a multifactorial treatment option to target photodamage along with pigmentation [19]. It is currently used for many applications within dermatology, as both a single-agent option and an adjunct to other treatment modalities, such as chemical peels [20]. On its own, ascorbic acid is water soluble and inherently unstable, and quickly and irreversibly oxidizes to 2,3-diketo-L-gulonic acid, resulting in a loss of its beneficial dermatologic properties [21]. Increased acidity of the vehicle, or combining ascorbic acid with vitamin E or ferulic acid, can help to increase its stability and shelf life [5]. Several vitamin C derivatives also exist on the market and are more stable at neutral pH compared to ascorbic acid. Some achieve this by converting to ascorbic acid when absorbed into the skin but penetrating the stratum corneum in another form to increase efficacy. While numerous studies support the role of vitamin C in treating hyperpigmentation, limited data exist regarding the comparative efficacy of vitamin C derivatives [22]. Our data show that consumers displayed no preference among the vitamin C derivatives, with no significant differences in average ratings for each active ingredient, which mirrors the lack of evidence for the relative performance of these derivatives.

In terms of product reviews, consumers for both retailers prioritized efficacy and cosmetic elegance, which mirrors our previous work assessing consumer preferences for tinted sunscreens [23]. The critical comments for products sold at Sephora more frequently mentioned cosmetic elegance compared to those for Amazon products. As Sephora is a large-scale beauty retailer with a significantly higher price point for skin care products, consumers may be expecting features beyond functionality, such as the ability to layer under makeup. While Amazon offers a wider range of products with a lower average price point, a prior study raised concerns regarding labeling and active ingredients of cosmeceutical products sold on the platform [24].

Our study is limited by including information from two retailers and only highly rated products. Derivatives not included in this study may be uncommonly used or more commonly found in less well-rated products. In addition, response bias is present as only a small proportion of consumers submit reviews, limiting the generalizability of our findings. Furthermore, there was no way to stratify consumers' comments based on the level of hyperpigmentation that they were experiencing prior to utilizing products and the remainder of their skincare regime is also not known. As a result, we are unable to comment on which formulations work best for varying levels of hyperpigmentation, and the data collected may not be able to adequately assess true efficacy when used as stand-alone products.

## Conclusions

Overall, our study shows that among vitamin C products on Amazon and Sephora that were highly rated, consumers displayed no preference for vitamin C derivatives in these products. This could mean that consumers are unable to distinguish between the effects of differing vitamin C derivatives or that current concentrations and formulations are roughly equal in the effect they are able to deliver. Therefore, patients may be utilizing products that are not correcting their underlying skin disorder, or are equivalent to other products currently on the market. It was also found that consumers prioritized efficacy and cosmetic elegance when purchasing these products. Regardless of the derivative used, it appears that consumers will rank products that are deemed more elegant over equivalent derivatives that have a less preferred texture or formulation. It is vital for dermatologists to inquire about, and remain aware of, OTC skincare products to counsel on best treatment practices and potential adverse effects due to market variety.
